# JAM3 promotes cervical cancer metastasis by activating the HIF-1α/VEGFA pathway

**DOI:** 10.1186/s12905-024-03127-7

**Published:** 2024-05-17

**Authors:** Jiali Peng, Yao Chen, Aijun Yin

**Affiliations:** 1 Department of Obstetrics and Gynecology, Qilu Hospital, Shandong University, 107 Wenhua Xi Road, Jinan, Shandong 250012 P. R. China; 2 Biomedical Sciences College & Shandong Medicinal Biotechnology Centre, Shandong First Medical University & Shandong Academy of Medical Sciences, Jinan, P. R. China

**Keywords:** JAM3, Cervical cancer, Metastasis, HIF-1α/VEGFA pathway

## Abstract

Cervical cancer is the fourth most common cancer and the leading cause of mortality among women worldwide. Tumor metastasis is an important cause of poor prognosis. Determining the exact mechanisms of metastasis and potential targeted therapies is urgently needed. Junctional adhesion molecule 3 (JAM3) is an important member of the TJ tight junction (TJ) family, and its biological function in cervical cancer needs to be further clarified. We found that JAM3 was highly expressed in cervical cancer patients with lymph node metastasis and that high expression of JAM3 promoted cervical cancer cell metastasis both in vitro and in vivo. In addition, overexpression of JAM3 induces epithelial–mesenchymal transition (EMT). Moreover, silencing JAM3 suppressed cervical cancer cell migration and invasion in vitro. Finally, JAM3 overexpression activated the HIF-1α/VEGFA pathway. In conclusion, our results suggested that JAM3 promotes cervical cancer cell migration and invasion by activating the HIF-1α/VEGFA pathway. JAM3 may be a promising biomarker and effective therapeutic target for cervical cancer.

**Supplementary Information:**

The online version contains supplementary material available at 10.1186/s12905-024-03127-7.

## Introduction

Cervical cancer is preventable; however, it is the fourth most common cancer and a leading cause of mortality among women worldwide. In 2020, over 600,000 new cases of cervical cancer were identified globally, representing 6.5% of all newly diagnosed cancers in women globally [1]. In 2020, approximately 90% of cervical cancer deaths globally occurred in low- and middle-income countries (LMICs) [1]. There have been great advances in the diagnostic and screening methods for cervical cancer in recent years, but the effectiveness of treatments for cervical cancer has not radically changed [2]. For primary prevention, the human papillomavirus (HPV) vaccine protects against infection or at least reduces the persistence of HPV infection [3]. Screening is performed through cervical smears (Pap tests) and HPV DNA tests, which are secondary prevention methods [4, 5]. Finally, tertiary prevention aims at the treatment of previously diagnosed lesions with the aid of surgery [5–9]. Surgery (with or without radiotherapy or chemotherapy) is a good option for small, localized recurrences without evidence of sidewall or distant disease [10]. Following distant lymph node metastasis, the 5-year overall survival rate decreases significantly [11]. For patients with distant recurrence/metastatic disease, platinum-based chemotherapy ± bevacizumab is recommended for chemotherapy-naive, medically fit patients. For patients with programmed death ligand 1 (PD-L1)-positive tumors, the addition of pembrolizumab to platinum-based chemotherapy ± bevacizumab is recommended [12]. Hence, further study of the molecular mechanisms of cervical cancer metastasis and identification of new therapeutic targets for cervical cancer are highly important.

In cancer, cell–cell adhesion and migration are essential processes that occur during the early stages of metastasis. Tight junction (TJ) proteins play well-established roles in tumor cell adhesion, polarity, invasion and migration [13]. Previous landmark studies have demonstrated that the lack or loss of TJ-based cell adhesion and epithelial barrier function increases cell permeability, leading to increased tumor cell invasion, dissemination, and metastasis [14, 15]. However, an increasing number of studies have suggested that TJ proteins may not function as tumor suppressors but rather accelerate tumor progression, suggesting that TJ proteins function in a context-dependent manner in cancer [16].

JAM3 is an important TJ protein. However, the function of JAM3 in tumors remains unclear. In a model of ovarian cancer, knockout of JAM3 in endothelial cells resulted in reduced pericyte coverage and increased vascular leakage, leading to longer mouse survival [17]. In addition to binding to JAM3 on endothelial cells, JAM3 also binds to JAM-B, which stimulates tumor cell metastasis and invasion [18, 19]. JAM3 methylation is an early detection and prognostic marker of esophageal cancer and suppresses esophageal cancer growth both in vitro and in vivo by inhibiting Wnt signalling [20]. The methylation level of JAM3 increases gradually with the severity of cervical lesions, suggesting that methylated JAM3 may be involved in the occurrence and development of cervical cancer [21]. However, the exact role of JAM3 in cervical cancer remains unclear. In this study, we found that JAM3 promoted cervical cancer cell migration and invasion both in vitro and in vivo. In addition, we found that JAM3 promotes cervical cancer metastasis by activating the HIF-1α/VEGFA pathway. Thus, JAM3 has the potential to be a new diagnostic biomarker and therapeutic target in patients with cervical cancer.

## Materials and methods

### Patients and tissue samples

Cervical cancer patients who underwent gynecological surgical excision at the Qilu Hospital of Shandong University were included in the study. A total of sixteen cervical cancer patients were included in the study. Based on pathological findings, all patients were classified as having lymph node metastasis or not having lymph node metastasis. The tissues were evaluated by two pathologists who were blinded to the clinical data. The basic information of the two groups of patients is provided in Table 1.

**Table 1:** Basic information of the two groups of patients included in the present study

Age	FIGO stage	Histologic subtypes	Lymph node metastasis
47	IB2	Squamous	No
23	IB2	Squamous	No
72	IB2	Squamous	No
39	IB3	Squamous	No
51	IB3	Squamous	No
22	IB3	Squamous	No
54	IB2	Squamous	No
31	IB3	Squamous	No
34	IB1	Squamous	No
35	IIA2	Squamous	No
58	IB3	Squamous	No
39	IIIC1	Squamous	Yes
60	IIIC1	Squamous	Yes
51	IIIC1	Adenocarcinoma	Yes
61	IIIC1	Squamous	Yes
45	IIIC1	Squamous	Yes

### Cell lines and cell culture

HeLa (SCSP-504), CaSki (BFN60700201), Siha (TCHu113), H8 (BFN607200572), and HEK293T (BFN60810479) cells were purchased from the Cell Bank of Type Culture Collection of the Chinese Academy of Sciences (Shanghai, China) and ATCC. All cell lines were cultured in DMEM supplemented with 10% fetal bovine serum (FBS), and the culture medium was supplemented with 1% penicillin/streptomycin. A humidified 37 °C incubator with 5% CO2 was used to culture all cells.

### Antibodies and reagents

Antibodies against JAM-C (Santa Cruz, sc-80134, 1:100), HIF-1α (Abcam, ab179483, 1:1000), β-actin (Sigma‒Aldrich, A5441, 1:5000), VEGFA (Proteintech, 66828–1-Ig, 1:1000), E-cadherin (Proteintech, 20874–1-AP, 1:1000), Snail (Proteintech, 13099–1-AP, 1:500), vimentin (Proteintech, 10366–1-AP, 1:1000), and Slug (Santa Cruz, sc-166476, 1:100) were used.

### RNA isolation and RT‒qPCR

Total RNA was isolated from the cells using TRIzol reagent (15596018, Invitrogen), and reverse transcription was performed using the PrimeScript RT reagent Kit (RR037A, TaKaRa, Kyoto, Japan). Real-time quantitative PCR (RT‒qPCR) was performed according to the instructions of SYBR Premix Ex Taq (RR420A, TaKaRa) using the 7900HT Fast Real-Time PCR System (Applied Biosystems, Waltham, MA, USA). β-Actin served as an endogenous control. The 2^−ΔΔCT^ method was used to analyse the relative expression of the targets. JAM3 forward primer: 5′-AGCCAATCCCAGATTTCGCAA-3′; reverse primer: 5′-TGAACAGCAGTGAACACCAAAG-3′; β-actin forward primer: 5′-AGTTGCGTTACACCCTTTC-3′; reverse primer: 5′-CCTTCACCGTTCCAGTTT-3′.

### Protein extraction and Western Blotting (WB)

Tissues and cells were lysed in radioimmunoprecipitation assay (RIPA) buffer (Beyotime Institute of Biotechnology, China) supplemented with PMSF (1%) and NaF (1%). Cell supernatants were collected after centrifugation at 12,000 rpm for 5 min. The protein concentration was calculated using a BCA Protein Assay Kit (Merck Millipore, 71287). A total of 40–80 µg of protein was separated via 8–15% gradient SDS‒PAGE and transferred onto PVDF membranes (Merck Millipore, Burlington, MA, USA). Next, the membrane was incubated with primary antibody at 4 °C overnight. The protein bands were then incubated with secondary antibodies and detected using an enhanced chemiluminescence (ECL) system (GE Healthcare). β-Actin was used as an endogenous control.

### Immunohistochemical (IHC) staining

Slides with cervical cancer tissues were sectioned from the paraffin-embedded TMAs. Antigen retrieval was performed by microwave heating in citrate buffer (pH 6.0) or EDTA (pH 9.0). The slides were incubated with 3% hydrogen peroxide for 20 min and blocked with goat serum for 30 min. Primary antibody was added, and the slides were incubated for 16 h at 4 °C. The appropriate secondary antibody was then added, and the sections were incubated for another 1 h. Finally, the sections were stained with a DAB detection system. The final degree of immunostaining was evaluated based on the extent and intensity of the staining.

### MTT assay

The 3-[4,5-dimethylthiazol-2-yl]-2,5-diphenyl tetrazolium bromide (MTT) assay was used to measure cell proliferation. Cells were seeded in 96-well plates at a concentration of 3000 cells per well. Before the absorbance was measured, 20 µL of MTT was added to each well, the plate was incubated for 4 h, and the supernatant was replaced with 100 μL of DMSO. The absorbance at 490 nm was measured using a microplate reader.

### Colony formation assay

Single-cell suspensions of cells were plated in six-well plates, and the medium was changed every three days. We used methanol to fix the cells and 0.6% crystal violet to stain them. The number of colonies was counted using ImageJ 1.52a software.

### Lentiviral infection and RNA interference

The JAM3 overexpression plasmid was obtained from GeneChem (Shanghai, China). Lentiviral vectors were packaged into HEK293T cells using psPAX2 and pMD2. G to produce lentiviral particles. Cervical cancer cell lines were generated by infection with lentiviral particles for 24 h and repeated infection for 24 h, followed by selection with puromycin (2 µg/ml) for 10 days to obtain stably transfected cell lines.

Specific JAM3 siRNAs (si JAM3#1:5′-GAGAGACUCAGCCCUUUAUTT-3′; si JAM3#2:5′-GCUACUUCAUCAACAAUAATT -3′) and a negative control siRNA (siNC: 5′-UUCUCCGAACGUGUCACGUTT-3′) were purchased from GenePharma (Shanghai, China). Cancer cells at an appropriate confluence were transfected with siJAM3 or NC using Lipofectamine 2000 according to the manufacturer’s instructions (11,668–019, Invitrogen).

### Invasion and migration assays

Invasion and migration assays were performed in 24-well transwell chambers (BD Biosciences, USA) with 8 μm pores coated with or without diluted Matrigel (BD Biosciences, USA). Cervical cancer cells (1 × 10^5^) were seeded into the upper compartment of the chamber, and culture medium supplemented with 20% FBS was added to the lower compartment. After incubation at 37 °C for 12 to 72 h, depending on the cell line, cells that penetrated through the membrane were fixed with methanol and stained with 0.6% crystal violet.

### Apoptosis assays

Apoptosis was quantified using annexin V-APC and 7-AAD. Treated cells were washed with PBS and digested with trypsin. The cells were then rinsed with PBS and resuspended in 1X binding buffer (556547, BD Biosciences, Franklin Lakes, NJ, USA). The cell suspension was stained and incubated with 5 µL of allophycocyanin (APC)-annexin V for 25 min and 5 µL of 7-aminoactinomycin D (7-AAD) for 15 min in the dark at room temperature. APC- and/or 7-AAD-positive cells were analysed using a flow cytometer (Beckman Coulter CytoFLEX, CA, USA).

### Xenograft tumor model

For the in vivo nude mouse metastasis assays, 200 µL suspensions of 1 × 10^6^ HeLa cells were used for tail vein injection, cells (5 × 10^5^ in 100 µL of PBS) were injected into the lateral tail veins 7 or 8 weeks later, and the lung tissues were removed and fixed in 10% formalin.

### Hematoxylin and Eosin (HE) staining

Slides with lung tissue were sectioned from paraffin-embedded TMAs. After dewaxing and hydration, the slides were stained with hematoxylin–eosin, sealed, and photographed using a full-section scanner.

### Statistical analysis

All data from three independent assays are shown as the mean ± standard error (SEM). Comparisons between two groups and more than two groups were performed using Student’s t test and one-way ANOVA, respectively, with GraphPad Prism 8.00 (GraphPad Software, La Jolla, CA, USA). Images were processed using Adobe Photoshop CC 2019 (Adobe, San Jose, CA, USA). A *p* value of < 0.05 was considered to indicate statistical significance (*P < 0.05, **P < 0.01, ***P < 0.001, and ****P < 0.0001).

## Results

### JAM3 is highly expressed in cervical cancer patients with lymph node metastasis

**Fig. 1:**
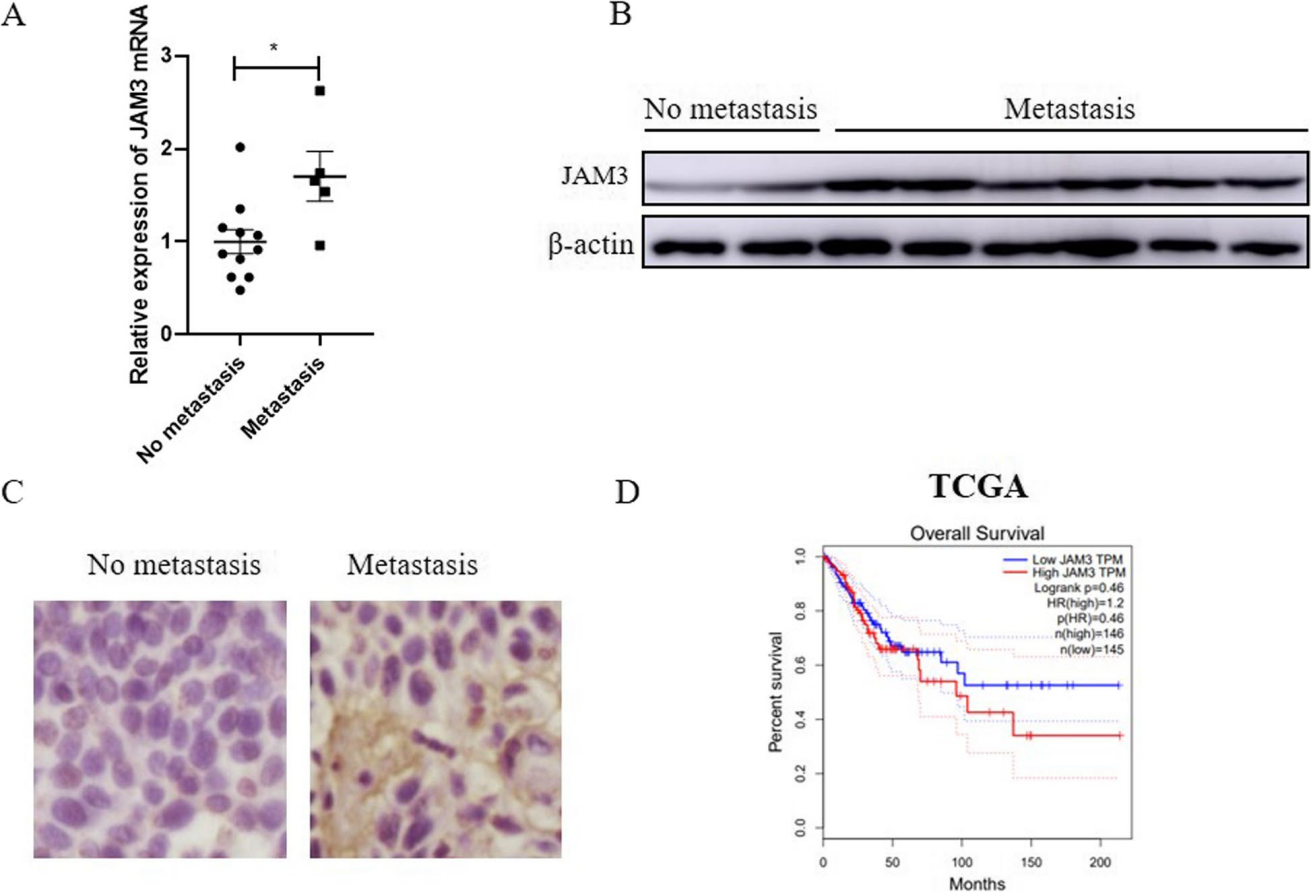
JAM3 is highly expressed in cervical cancer patients with lymph node metastasis. **A**-**B** The mRNA and protein levels of JAM3 in cervical cancer patients with or without lymph node metastasis. The full-length blots are presented in Supplementary Fig. 1A. **C** Representative IHC staining (400 ×) of JAM3 in cervical cancer tissues with or without lymph node metastasis. **D** TCGA database analysis showing the correlation between the mRNA expression of JAM3 and overall survival in cervical cancer patients

To verify the expression levels of JAM3 in lymph node-positive and lymph node-negative patients with cervical cancer, JAM3 mRNA and protein expression were detected by RT‒qPCR and western blotting in fresh-frozen tissues, and we found that JAM3 was frequently upregulated in cervical cancer patients with lymph node metastasis (Fig. 1A‒B, *p* = 0.0171). To further confirm the above results, immunohistochemistry (IHC) was conducted to assess the expression profile of JAM3 in cervical cancer. JAM3-positive staining was located in the cytoplasm, and JAM3 expression in lymph node-positive patients with cervical cancer was significantly greater than that in lymph node-negative patients (Fig. 1C). The TCGA database also revealed that the upregulation of JAM3 in cervical cancers correlated with poorer prognosis (Fig. 1D).

### JAM3 depletion suppresses cervical cancer cell migration and invasion in vitro

**Fig. 2:**
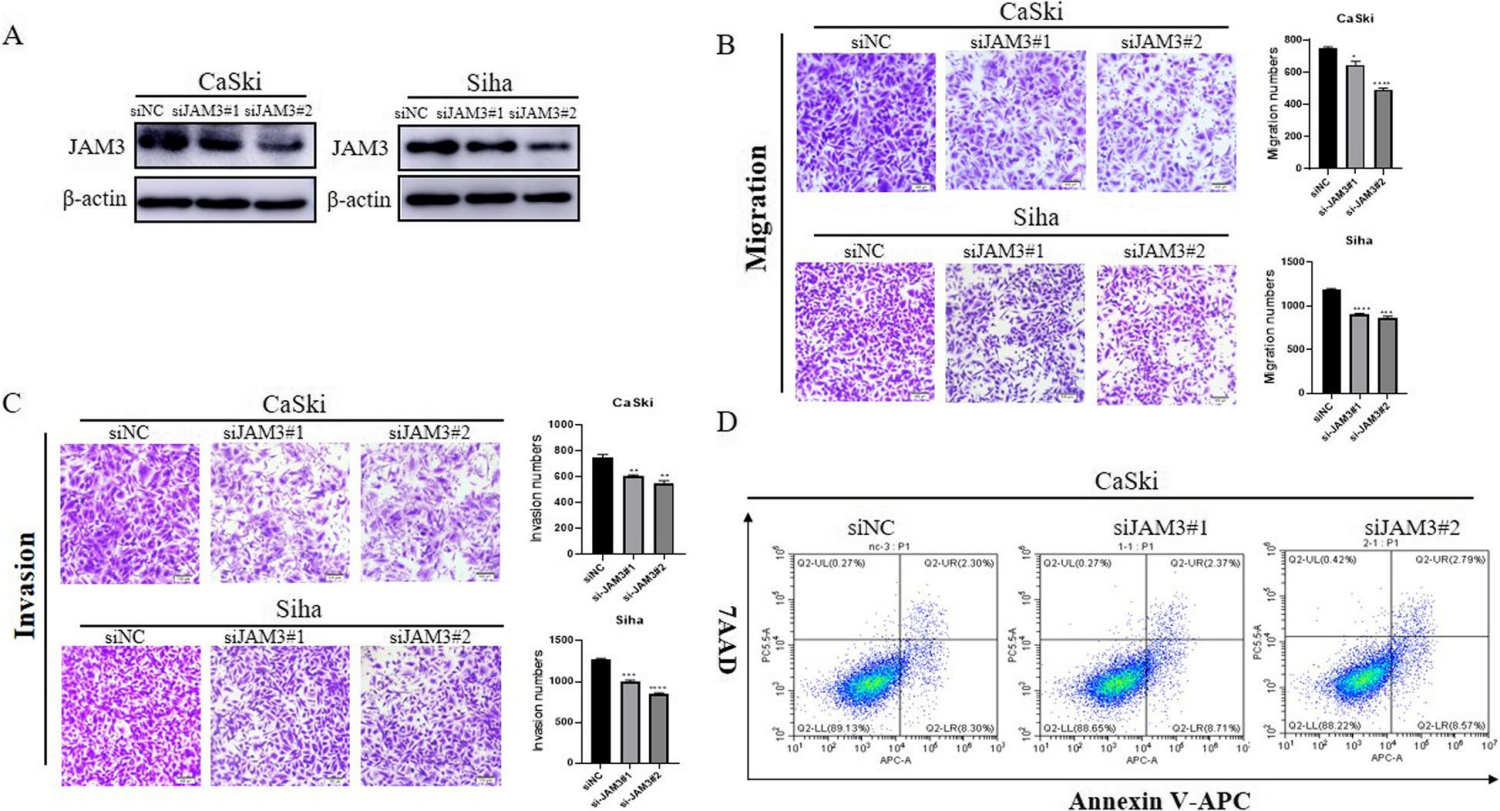
JAM3 depletion suppresses cervical cancer cell migration and invasion in vitro. **A** The protein level of JAM3 in cervical cancer cells after JAM3 depletion. The full-length blots are presented in Supplementary Fig. 1D. **B-C** Transwell assays showing the migration and invasion capacity of cervical cancer cells upon JAM3 depletion. **D** Flow cytometry assays were performed to analyse apoptosis

We generated two kinds of small interfering RNAs (siRNAs) to downregulate JAM3 expression. The JAM3 knockdown efficiency was verified by western blotting (Fig. 2A). Migration and invasion assays were performed, and we found that JAM3 depletion suppressed cervical cancer cell migration and invasion in vitro (Fig. 2B-C, *p* = 0.0142, *p* < 0.0001, *p* < 0.0001, *p* = 0.0002, *p* = 0.0024, *p* = 0.0027, *p* = 0.0004, *p* < 0.0001). An apoptosis assay was performed to determine whether JAM3 knockdown affected cervical cancer cells undergoing apoptosis. As shown in Fig. 2D, there were no differences among the three groups. These results indicate that JAM3 depletion suppresses cervical cancer cell migration and invasion but not apoptosis.

### JAM3 promotes cervical cancer cell migration and invasion in vitro

**Fig. 3:**
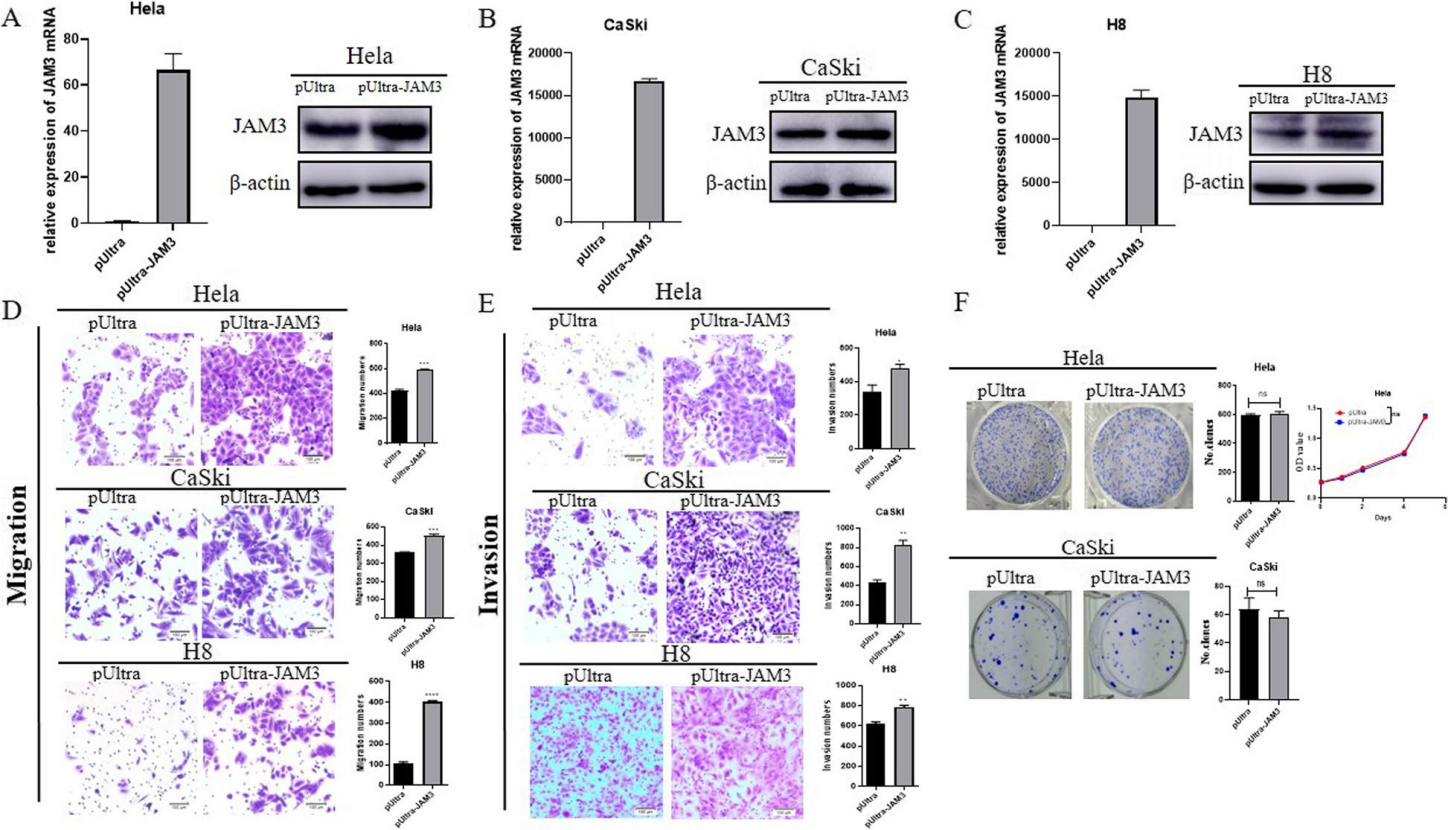
JAM3 promotes cervical cancer cell migration and invasion in vitro. **A**-**C** The mRNA and protein levels of JAM3 in cervical cancer cells after JAM3 overexpression. The full-length blots are presented in Supplementary Fig. 1B. **D-E** Transwell assays showed the migration and invasion capacity of cervical cancer cells upon JAM3 overexpression. **F** Proliferation curves and clonogenic assays were used to evaluate the effect of JAM3 overexpression on the proliferation of HeLa and CaSki cells

To explore the role of JAM3 in the development and progression of cervical cancer, HeLa, CaSki, and H8 cells were stably transfected with pUltra-JAM3 to overexpress JAM3. RT‒qPCR and WB showed that the mRNA and protein expression levels of these genes were significantly increased after JAM3 overexpression (Fig. 3A‒C, *p* < 0.0001, *p* < 0.0001, *p* < 0.0001). Since increased levels of JAM3 in lymph node-positive patients with cervical cancer were significantly related to metastasis, we hypothesized that JAM3 promotes the invasive capacity of cervical cancer cells. We measured migration and invasion abilities using a transwell assay, and our results showed that upregulation of JAM3 increased the migration and invasion capacity of cervical cancer cells (Fig. 3D-E, *p* = 0.0003, *p* = 0.0006, *p* < 0.0001, *p* = 0.0439, *p* = 0.0020, *p* = 0.0035). To investigate whether JAM3 could influence the proliferation of cervical cancer cells, colony formation and MTT assays were conducted, and the results showed that JAM3 did not influence the proliferation of cancer cells (Fig. 3F, *p* = 0.8008, *p* = 0.5469, *p* = 0.9646).

### JAM3 promotes cervical cancer cell invasion in vivo and induces epithelial–mesenchymal transition (EMT)

**Fig. 4:**
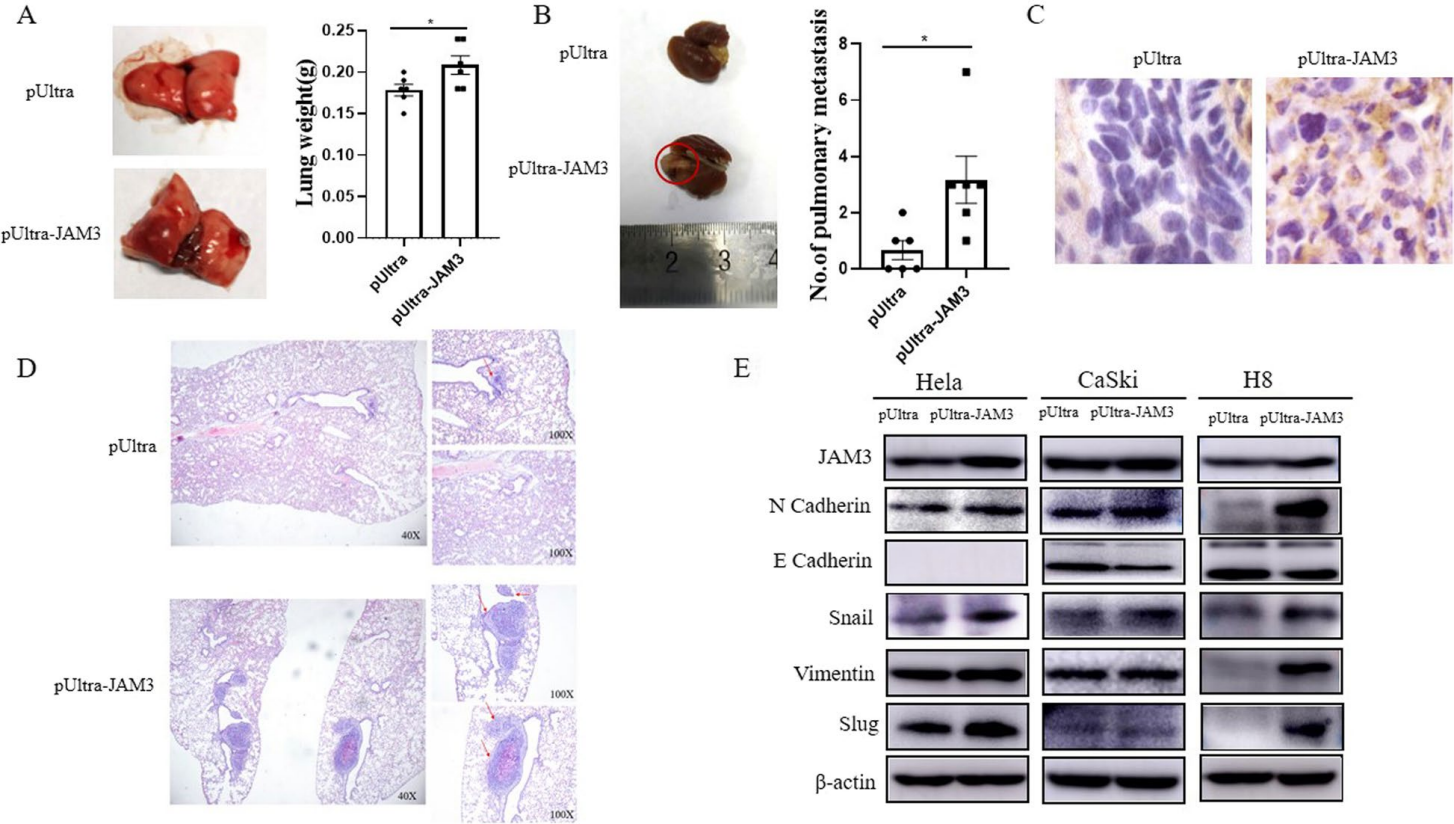
JAM3 promotes cervical cancer cell invasion in vivo and induces epithelial–mesenchymal transition (EMT). **A-B** The lung weight was measured, and the number of lung metastasis nodules was calculated after JAM3 overexpression. **C-D** Representative images of lungs and HE staining and IHC staining (400 ×) of tissues isolated from mice that received a tail vein injection. **E** EMT-related markers were detected by western blot in HeLa, CaSki and H8 cells overexpressing JAM3. The full-length blots are presented in Supplementary Fig. 1C

Next, we evaluated the metastasis-promoting potential of JAM3 in a lung metastasis model using tail vein injection. JAM3 overexpression increased the lung weight compared to that in the control group (Fig. 4A, *p* = 0.0453). The number of lung metastatic nodules also increased, as shown in Fig. 4B (*p* = 0.0193). Moreover, HE staining suggested that the size of the lung metastasis nodules significantly increased after JAM3 overexpression (Fig. 4C). IHC analysis of JAM3 further confirmed the overexpression of JAM3 (Fig. 4D). Since JAM3 promoted cervical cancer cell invasion in vitro and in vivo, we assessed the expression of EMT markers by western blotting, and the results showed that upregulated expression of JAM3 in HeLa, CaSki, and H8 cells increased the expression of mesenchymal phenotype markers (N-cadherin, Snail, Vimentin, and Slug) and reduced the expression of an epithelial phenotype marker (E-cadherin) (Fig. 4E). These results indicated that JAM3 promotes cervical cancer cell metastasis.

### JAM3 promotes cervical cancer cell migration and invasion by activating the HIF-1α/VEGFA pathway

**Fig. 5:**
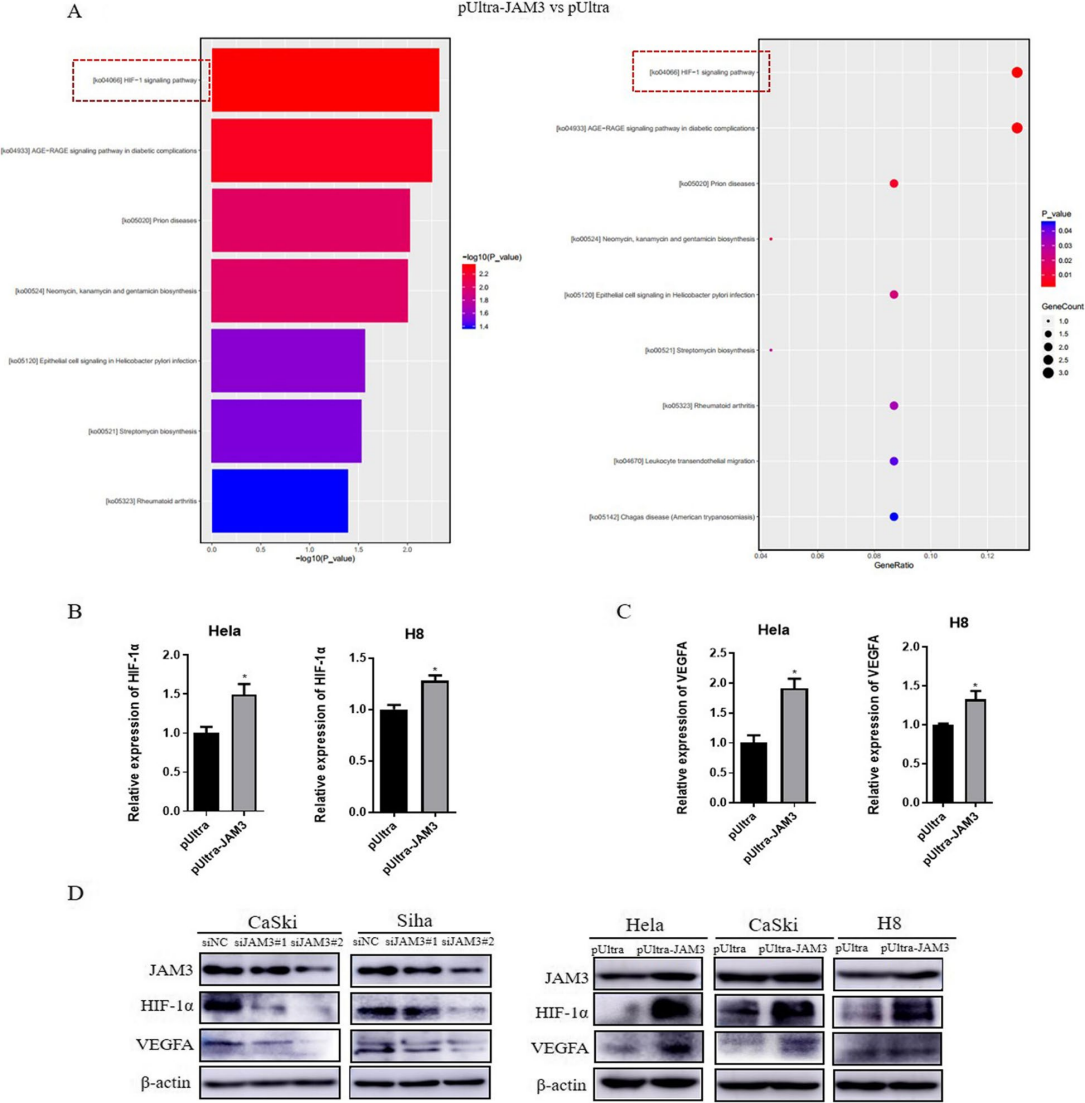
JAM3 promotes cervical cancer cell migration and invasion by activating the HIF-1α/VEGFA pathway. **A** NGS analysis of JAM3-affected signalling pathways. **B-C** The mRNA levels of HIF-1α and VEGFA in cervical cancer cells after JAM3 overexpression. **D** The protein levels of HIF-1α and VEGFA in cervical cancer cells after JAM3 overexpression or depletion. The full-length blots are presented in Supplementary Fig. 1E

Next-generation sequencing (NGS) was performed in HeLa cells transfected with pUltra-JAM3 or pUltra (*n* = 3) to clarify the potential mechanism by which JAM3 promotes cervical cancer cell migration and invasion. As shown in Fig. 5A, the HIF-1 signalling pathway was activated. RT‒qPCR revealed that the mRNA expression of HIF-1α and VEGFA was significantly upregulated after JAM3 overexpression in HeLa and H8 cells (Fig. 5B-C, *p* = 0.0331, *p* = 0.0140, *p* = 0.0103, *p* = 0.0296). The protein expression levels of HIF-1α and VEGFA were further verified after JAM3 overexpression or depletion (Fig. 5D). These results demonstrated that JAM3 promotes cervical cancer progression by activating the HIF-1α/VEGFA pathway.

## Discussion

Cervical cancer remains prevalent in women worldwide, and there are limited treatment options for patients with locally advanced and metastatic cervical cancer. JAM3 is a member of the immunoglobulin (Ig)-like JAM family. However, the role of JAM3 in tumors is still unclear. JAM3 functions as a novel tumor suppressor and is inactivated by DNA methylation in colorectal cancer [22]. On the other hand, JAM3 has been reported to be expressed in melanoma and endothelial cells and consequently promotes lung metastasis in melanoma by mediating the transendothelial migration of melanoma cells [23]. In addition, JAM3 promotes lymphangiogenesis and nodal metastasis in non-small cell lung cancer, and this cancer-promoting mechanism is driven by it, which contributes to VEGF-C expression in cancer cells [24]. The expression of JAM-C promotes metastasis by enhancing both the adhesion of cancer cells to extracellular matrices and the subsequent invasion of HT1080 human fibrosarcoma cells [25]. Consistent with the above findings, we found that JAM3 was highly expressed in cervical cancer patients with lymph node metastasis and that high expression of JAM3 promoted cervical cancer cell metastasis both in vitro and in vivo.

Patients with persistent/recurrent disease outside the pelvis were classified as patients with metastatic cervical cancer. Thirteen percent of patients with cervical cancer are diagnosed at an advanced stage. The 5-year survival rate for patients with metastatic cervical cancer is 16.5%, while that for patients with localized cervical cancer is 91.5% [26]. Since tumor metastasis is associated with poorer prognosis, it is important to elucidate the molecular mechanisms underlying the metastasis of cervical cancer. EMT is a process by which epithelial cells acquire a mesenchymal stem phenotype. This process is involved in many fundamental processes, including embryonic evolution, tissue formation, wound healing, and tissue fibrosis. Moreover, EMT can affect tumor cell growth, drug resistance, and tumor proliferation. Importantly, a large amount of literature has established that EMT plays a role in the metastasis of tumor cells [27, 28]. In line with these findings, our results suggest that overexpression of JAM3 induces EMT. As a consequence, JAM3 overexpression promotes CC cell migration and invasion.

HIF-1α is a hypoxia-responsive factor that responds to hypoxia by activating the master regulator of the transcription of many genes and participates in cell energy metabolism, angiogenesis, proliferation, and apoptosis [29]. HIF-1α is upregulated in many tumors. Studies have shown that HIF-1α acts as an oncogene and participates in tumor growth and metastasis [30]. Increasing evidence has demonstrated that VEGFA is an important downstream factor of HIF-1 [29]. Accumulating evidence has shown that VEGFA plays a key role in tumor angiogenesis and vascular mimicry [31, 32]. In this study, we found that upon overexpression of JAM3, the mRNA and protein expression of HIF-1α and VEGFA were significantly upregulated. Therefore, JAM3 overexpression activates the HIF-1α/VEGFA pathway. Considering the above findings, we demonstrated that JAM3 promotes cervical cancer cell migration and invasion by activating the HIF-1α/VEGFA pathway.

In summary, our study demonstrated that JAM3 is highly expressed in cervical cancer patients with lymph node metastasis and that high expression of JAM3 promotes cervical cancer cell metastasis both in vitro and in vivo. EMT was also activated upon overexpression of JAM3. JAM3 depletion suppresses cervical cancer cell migration and invasion in vitro. In addition, JAM3 overexpression activated the HIF-1α/VEGFA pathway. Since angiogenesis plays a significant role in tumor metastasis, our research may provide directions for the suppression of tumor metastasis. However, the mechanism by which JAM3 activates the HIF-1 signalling pathway is still unclear, and this topic needs to be studied in the future. In conclusion, our results suggested that JAM3 promotes cervical cancer cell migration and invasion by activating the HIF-1α/VEGFA pathway. Efforts should be made to establish methods that can directly and effectively target JAM3 in the near future.

### Euthanasia/sacrifice methods

Pentobarbital sodium was intraperitoneally injected at a dose of 150–300 mg/kg into the mice. All the treatments were performed gently, and all efforts were made to minimize animal suffering.

### Supplementary Information

Supplementary Material 1.

Supplementary Material 2.

## Data Availability

All data in this study are available from the corresponding author upon reasonable request.
