# Supplementary figures and images for: JAM3 promotes cervical cancer metastasis by activating the HIF-1α/VEGFA pathway
Source: BMC Womens Health. 2024 May 17;24:293. doi: 10.1186/s12905-024-03127-7 (PMC11100123; doi:10.1186/s12905-024-03127-7)

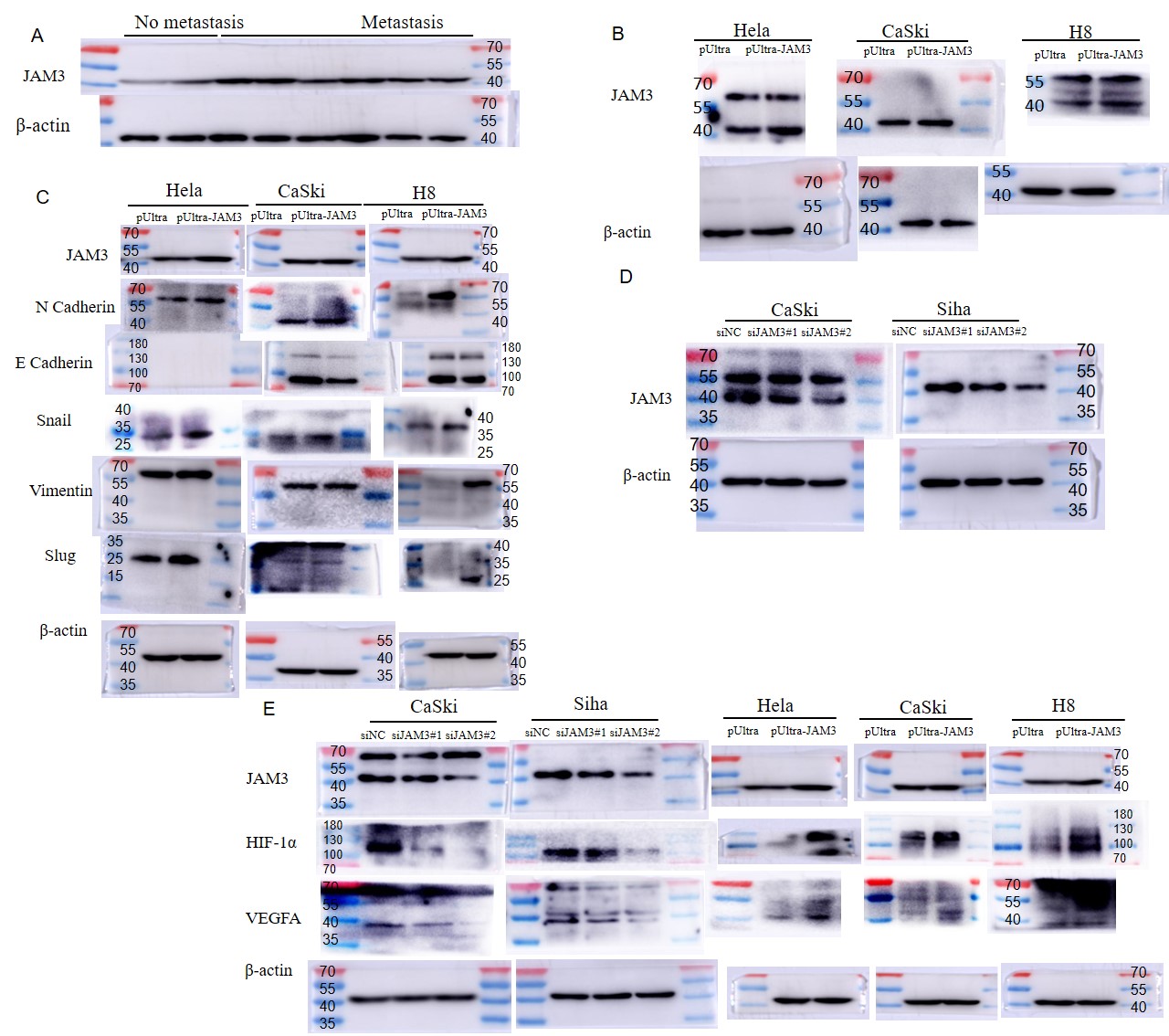

Supplement: Supplementary file 1 — Supplementary Material 1. [file 12905_2024_3127_MOESM1_ESM.jpg]

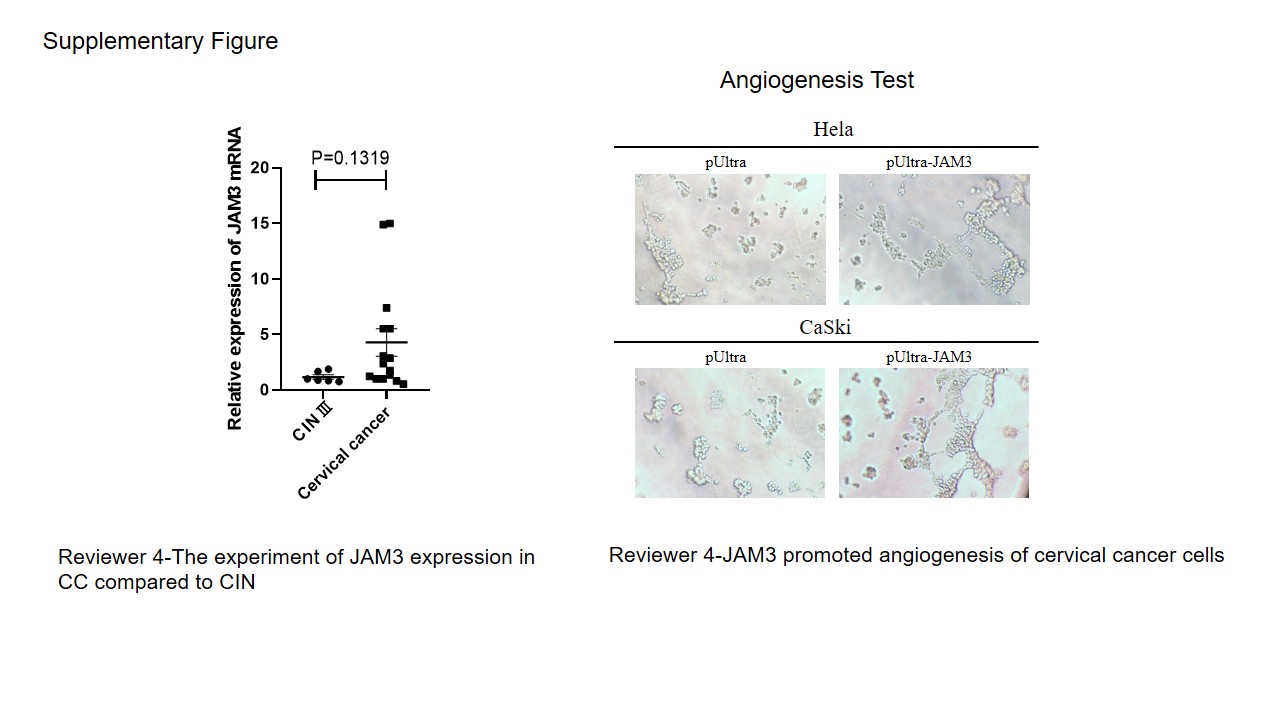

Supplement: Supplementary file 2 — Supplementary Material 2. [file 12905_2024_3127_MOESM2_ESM.jpg]
